# Role of Color Doppler and Microvascular Flow Imaging in the Assessment of Mesenteric Lymph Nodes in Pediatric Patients

**DOI:** 10.7759/cureus.113126

**Published:** 2026-07-21

**Authors:** Manik Sharma, Madan Mohan Babu L, Suresh A

**Affiliations:** 1 Radio Diagnosis, Vydehi Institute of Medical Sciences and Research Centre, Bengaluru, IND

**Keywords:** color doppler, intranodal vascularity, mesenteric lymphadenopathy, microvascular flow imaging, pediatric ultrasonography

## Abstract

Introduction

Pediatric mesenteric lymph nodes are frequently evaluated using ultrasonography in children presenting with abdominal pain. While Color Doppler is routinely used to assess intranodal vascularity, its limited ability to detect slow-flow microvascular signals may reduce diagnostic sensitivity. Microvascular Flow Imaging (MVFI) has emerged as an advanced Doppler technique capable of visualizing low-velocity blood flow with greater sensitivity. The present study aimed to evaluate the role of Color Doppler and Microvascular Flow Imaging in the assessment of mesenteric lymph nodes in pediatric patients and to compare their performance in detecting intranodal vascularity, vascularity grading, lymph node morphology, and diagnostic agreement.

Materials and methods

This prospective observational study was conducted in the Department of Radio Diagnosis at Vydehi Institute of Medical Sciences and Research Centre, Bengaluru, India, between May 2024 and December 2025. A total of 90 pediatric patients aged 2-15 years with clinically suspected mesenteric lymphadenopathy underwent grayscale ultrasonography, Color Doppler, and MVFI. The largest representative mesenteric lymph node was evaluated for morphological characteristics, vascularity, and vascularity grading. Diagnostic agreement was assessed using Cohen's kappa analysis, and the diagnostic performance of Color Doppler was evaluated using MVFI as the reference standard.

Results

The majority of patients belonged to the 6-10-year age group (43 {47.8%}), with a slight male predominance (51 {56.7%}). Abdominal pain was the most common presenting symptom (77 {85.6%}). Most lymph nodes demonstrated an oval morphology (79 {87.8%}) with preserved fatty hilum (80 {88.9%}). MVFI detected vascularity in 81 (90.0%) patients compared with 67 (74.4%) using Color Doppler (p = 0.004). Higher vascularity grades (G2/G3) were more frequently identified with MVFI, and lymph nodes demonstrating vascularity had significantly larger long-axis and short-axis diameters (p < 0.001). Color Doppler demonstrated a sensitivity of 82.7% (95% confidence interval {CI}: 72.7%-90.2%), specificity of 100.0% (95% CI: 66.4%-100.0%), diagnostic accuracy of 84.4% (95% CI: 75.3%-91.2%), and moderate agreement with MVFI (Cohen's κ = 0.52; p < 0.001).

Conclusion

Microvascular Flow Imaging demonstrated superior detection of intranodal vascularity compared with conventional Color Doppler and provided additional information for the characterization of mesenteric lymph nodes. When used as an adjunct to routine grayscale ultrasonography, MVFI may improve diagnostic confidence in children presenting with suspected mesenteric lymphadenopathy.

## Introduction

Mesenteric lymphadenopathy is a frequent finding in children presenting with abdominal pain and is commonly encountered during abdominal ultrasonography [1]. Although it is generally a benign and self-limiting condition, enlarged mesenteric lymph nodes may mimic acute appendicitis and other surgical causes of acute abdomen, posing a diagnostic challenge for clinicians [2]. The accurate identification and characterization of mesenteric lymph nodes are therefore essential to facilitate appropriate patient management, avoid unnecessary surgical interventions, and provide reassurance to patients and caregivers [3]. Ultrasonography has emerged as the first-line imaging modality for evaluating pediatric abdominal pain because of its wide availability, absence of ionizing radiation, noninvasive nature, and excellent diagnostic performance in children [4].

Conventional grayscale ultrasonography provides valuable information regarding the size, number, location, morphology, cortical echotexture, and fatty hilum of mesenteric lymph nodes [5]. The addition of Color Doppler imaging further enhances assessment by demonstrating intranodal vascularity, which may reflect the underlying inflammatory activity [1,5]. However, Color Doppler has inherent limitations in detecting slow-velocity blood flow within small-caliber intranodal vessels due to motion artefacts and the use of wall filters, potentially resulting in the underestimation of intranodal microvascular perfusion [6]. Consequently, subtle vascular changes associated with early or mild inflammatory processes may remain undetected using conventional Doppler techniques [7,8].

Microvascular Flow Imaging (MVFI) is an advanced Doppler-based ultrasound technique specifically developed to improve the visualization of low-velocity microvascular blood flow without the need for intravenous contrast agents [9,10]. By effectively suppressing tissue motion artefacts while preserving weak flow signals, MVFI enables the superior depiction of the microvascular architecture compared with conventional Color Doppler [11]. Recent studies have demonstrated its potential utility in evaluating superficial organs, inflammatory lesions, lymph nodes, thyroid abnormalities, breast lesions, and pediatric abdominal pathologies [9,12,13]. In mesenteric lymphadenopathy, the enhanced visualization of intranodal vascularity may improve lesion characterization, facilitate differentiation between reactive and clinically significant inflammatory lymph nodes, and increase diagnostic confidence during routine ultrasound examinations [14,15].

Despite the growing application of Microvascular Flow Imaging in pediatric imaging, comparative evidence evaluating its diagnostic performance against conventional Color Doppler for the assessment of mesenteric lymph nodes remains limited, particularly in the Indian pediatric population. We hypothesized that Microvascular Flow Imaging would demonstrate superior detection of intranodal vascularity and provide improved characterization of mesenteric lymph nodes compared with conventional Color Doppler. The present study was therefore undertaken to evaluate the role of Microvascular Flow Imaging in the assessment of mesenteric lymph nodes in pediatric patients.

## Materials and methods

This prospective observational study was conducted in the Department of Radio Diagnosis at Vydehi Institute of Medical Sciences and Research Centre, Bengaluru, Karnataka, India, over a period of 20 months from May 2024 to December 2025. The study was approved by the Institutional Ethics Committee of Vydehi Institute of Medical Sciences and Research Centre (approval number: VIEC/2023/APP/PG/55), and written informed consent was obtained from all patients or their parents/legal guardians, as appropriate, prior to enrolment in the study.

The sample size was calculated using the single population proportion formula:

\[ n=\frac{Z_{1-\alpha/2}^{2}\times p\times q}{d^{2}} \]

where n is the required sample size, Z is the standard normal deviate at the 95% confidence level (1.96), p is the expected prevalence of mesenteric lymphadenopathy (17.07%) reported by Sabal et al., q = 1 - p (82.93%), and d is the absolute precision (7.8%) [16]. Based on these assumptions, the calculated sample size was approximately 89.4, which was rounded up to 90 participants. Accordingly, a total of 90 pediatric patients fulfilling the predefined inclusion and exclusion criteria were enrolled in the study. Participants were recruited using consecutive sampling until the required sample size was achieved.

Inclusion criteria

Pediatric patients aged 2-15 years presenting with acute or recurrent abdominal pain and clinically suspected mesenteric lymphadenopathy who were referred for abdominal ultrasonography during the study period were included. Only patients who provided written informed consent through their parents or legal guardians prior to participation were enrolled.

Exclusion criteria

Patients with previously diagnosed malignant abdominal or lymphoproliferative disorders, a history of abdominal surgery within the preceding six months, congenital gastrointestinal anomalies, tuberculous or chronic granulomatous lymphadenopathy, or inadequate imaging quality due to poor patient cooperation or technical limitations or patients who refused to provide informed consent were excluded from the study.

All participants underwent comprehensive abdominal ultrasonography using the Samsung V6 and Samsung V8 (Samsung Medison, Seoul, South Korea) platforms, equipped with grayscale imaging, Color Doppler, and Microvascular Flow Imaging (MVFI). Examinations were performed using a high-frequency linear-array transducer (3-12 MHz), with a curvilinear transducer utilized when required to optimize the visualization of deeper abdominal structures. Initial grayscale ultrasonography was performed to identify mesenteric lymph nodes and assess their anatomical and morphological characteristics. The largest representative mesenteric lymph node identified in each patient was selected for detailed evaluation. The recorded grayscale parameters included anatomical location, long-axis diameter, short-axis diameter, long-to-short axis ratio, nodal shape (oval or rounded), cortical echotexture (homogeneous or heterogeneous), and the status of the central fatty hilum (preserved or absent) [5].

Following grayscale evaluation, Color Doppler imaging was performed using standardized settings with the optimization of pulse repetition frequency, wall filter, color gain, and color box size to maximize the visualization of intranodal vascularity while minimizing motion artefacts. Subsequently, the same lymph node was evaluated using Microvascular Flow Imaging (MVFI), an advanced Doppler technique designed to detect slow-flow microvascular perfusion by suppressing tissue motion artefacts while preserving low-velocity blood flow signals. MVFI examinations were performed using the manufacturer's dedicated microvascular imaging preset with a low wall filter, low pulse repetition frequency, optimized color gain below the background noise threshold, and minimal transducer pressure to maximize the visualization of low-velocity intranodal blood flow while minimizing motion artefacts [9-11]. Vascularity was graded on both imaging modalities using a four-grade classification: Grade G0 (no detectable vascularity), Grade G1 (minimal hilar vascularity), Grade G2 (moderate hilar vascularity), and Grade G3 (marked hilar and/or peripheral vascularity) [5]. All examinations were performed according to a standardized imaging protocol by radiologists experienced in pediatric ultrasonography. Before the commencement of the study, the imaging protocol and vascularity grading criteria were standardized through calibration sessions to ensure uniform image acquisition and interpretation, thereby minimizing interobserver variability and improving reproducibility.

The primary objective of the study was to compare the performance of Color Doppler with Microvascular Flow Imaging in detecting intranodal vascularity in mesenteric lymph nodes. Secondary outcome measures included the comparison of vascularity grades between the two imaging modalities, assessment of grayscale morphological characteristics of mesenteric lymph nodes, evaluation of the association between lymph node dimensions and vascularity, and determination of the diagnostic agreement between Color Doppler and Microvascular Flow Imaging. As histopathological confirmation was not routinely indicated or ethically justified in children with clinically suspected reactive mesenteric lymphadenopathy, Microvascular Flow Imaging was considered the reference standard for estimating the diagnostic performance of conventional Color Doppler [2,5].

The collected data were entered into Microsoft Excel (Microsoft Corp., Redmond, WA) and analyzed using IBM SPSS Statistics for Windows, version 26.0 (IBM Corp., Armonk, NY; released 2019). Continuous variables were expressed as mean ± standard deviation, whereas categorical variables were presented as frequency and percentage (N {%}). Comparisons between categorical variables were performed using the chi-square test, while continuous variables were compared using the independent samples t-test. Differences in paired vascularity grades (G0-G3) between Color Doppler and Microvascular Flow Imaging were analyzed using the Stuart-Maxwell test for marginal homogeneity. Diagnostic agreement between Color Doppler and Microvascular Flow Imaging was evaluated using Cohen's kappa coefficient. Sensitivity, specificity, positive predictive value, negative predictive value, and diagnostic accuracy with corresponding 95% confidence intervals (CI) were calculated using MVFI as the reference standard. No adjustment for multiple comparisons was performed because the analyses were prespecified and exploratory in nature. A p-value of <0.05 was considered statistically significant.

## Results

The study included 90 pediatric patients, with the highest proportion belonging to the 6-10-year age group (43, 47.8%), followed by 2-5 years (28, 31.1%) and 11-15 years (19, 21.1%). There was a slight male predominance, with 51 (56.7%) men and 39 (43.3%) women. Abdominal pain was the most common presenting symptom, observed in 77 (85.6%) patients, followed by fever in 41 (45.6%), vomiting in 34 (37.8%), loss of appetite in 25 (27.8%), and diarrhea in 18 (20.0%) patients (Table 1).

**Table 1:** Baseline Demographic and Clinical Characteristics of the Study Participants (n = 90) Data are presented as numbers (percentages) (N {%}). Descriptive statistics were used N: number of participants

Variable	Category	N (%)
Age group (years)	2-5	28 (31.1%)
	6-10	43 (47.8%)
	11-15	19 (21.1%)
Gender	Male	51 (56.7%)
	Female	39 (43.3%)
Presenting symptoms	Abdominal pain	77 (85.6%)
	Fever	41 (45.6%)
	Vomiting	34 (37.8%)
	Diarrhea	18 (20.0%)
	Loss of appetite	25 (27.8%)

Grayscale ultrasonography demonstrated that the largest mesenteric lymph node was most commonly located in the periumbilical region in 38 (42.2%) patients, followed by the right iliac fossa in 33 (36.7%) and the mesenteric root in 19 (21.1%) patients. Most lymph nodes exhibited an oval morphology in 79 (87.8%) patients, homogeneous cortical echotexture in 78 (86.7%), and a preserved fatty hilum in 80 (88.9%), indicating predominantly benign ultrasonographic features. The mean long-axis diameter was 16.4 ± 4.6 mm, the mean short-axis diameter was 6.8 ± 2.1 mm, and the mean long-to-short axis ratio was 2.42 ± 0.53 (Table 2).

**Table 2:** Grayscale Ultrasonographic Characteristics of Mesenteric Lymph Nodes (n = 90) Data are presented as numbers (percentages) (N {%}) or mean ± standard deviation (SD), as appropriate. Descriptive statistics were used mm: millimeters

Variable	Category	N (%)/Mean ± SD
Location of largest lymph node	Mesenteric root	19 (21.1%)
	Periumbilical region	38 (42.2%)
	Right iliac fossa	33 (36.7%)
Shape	Oval	79 (87.8%)
	Rounded	11 (12.2%)
Cortical echotexture	Homogeneous	78 (86.7%)
	Heterogeneous	12 (13.3%)
Fatty hilum	Preserved	80 (88.9%)
	Absent	10 (11.1%)
Long-axis diameter (mm)	Mean ± SD	16.4 ± 4.6
Short-axis diameter (mm)	Mean ± SD	6.8 ± 2.1
Long-to-short axis ratio	Mean ± SD	2.42 ± 0.53

Microvascular Flow Imaging demonstrated superior detection of intranodal vascularity compared with conventional Color Doppler. Vascularity was detected in 81 (90.0%) patients using Microvascular Flow Imaging compared with 67 (74.4%) patients using Color Doppler, whereas the absence of vascularity was observed in nine (10.0%) and 23 (25.6%) patients, respectively. The difference in vascularity detection between the two imaging modalities was statistically significant (McNemar χ² = 8.10; p = 0.004), indicating superior vascularity detection by MVFI (Table 3).

**Table 3:** Comparison of Color Doppler with Microvascular Flow Imaging Findings (n = 90) Data are presented as numbers (percentages) (N {%}). McNemar's chi-square (χ²) test was used for comparison. A p-value of <0.05 was considered statistically significant

Imaging Finding	Color Doppler	Microvascular Flow Imaging	McNemar χ²	P-value
Vascularity detected	67 (74.4%)	81 (90.0%)	8.10	0.004
No vascularity detected	23 (25.6%)	9 (10.0%)		

A comparison of vascularity grades showed that Microvascular Flow Imaging identified a greater proportion of patients with higher vascularity grades than Color Doppler. Grade G3 vascularity was observed in 27 (30.0%) patients using Microvascular Flow Imaging compared with 13 (14.4%) patients using Color Doppler, while Grade G0 was identified in only nine (10.0%) patients with Microvascular Flow Imaging compared with 23 (25.6%) patients using Color Doppler. Grade G2 vascularity was detected in 36 (40.0%) patients with Microvascular Flow Imaging and 30 (33.3%) patients with Color Doppler, whereas Grade G1 vascularity was observed in 18 (20.0%) and 24 (26.7%) patients, respectively. The overall distribution of vascularity grades differed significantly between the two imaging modalities according to the Stuart-Maxwell test for marginal homogeneity (p < 0.001) (Table 4).

**Table 4:** Comparison of Vascularity Grades on Color Doppler and Microvascular Flow Imaging (n = 90) Data are presented as numbers (percentages) (N {%}). The Stuart-Maxwell test for marginal homogeneity was used for comparison. A p-value of <0.05 was considered statistically significant

Vascularity Grade	Color Doppler	Microvascular Flow Imaging	Stuart-Maxwell test	P-value
G0 (no vascularity)	23 (25.6%)	9 (10.0%)	13.20	<0.001
G1 (minimal hilar vascularity)	24 (26.7%)	18 (20.0%)		
G2 (moderate hilar vascularity)	30 (33.3%)	36 (40.0%)		
G3 (marked hilar/peripheral vascularity)	13 (14.4%)	27 (30.0%)		

Patients demonstrating vascularity on Microvascular Flow Imaging had significantly larger lymph node dimensions than those without detectable vascularity. The mean long-axis diameter was 17.1 ± 4.4 mm among 81 (90.0%) patients with vascularity compared with 11.5 ± 2.8 mm among nine (10.0%) patients without vascularity (t = 4.18; p < 0.001). Similarly, the mean short-axis diameter was significantly greater in patients with vascularity (7.1 ± 1.9 mm) than in those without vascularity (4.6 ± 1.3 mm) (t = 4.73; p < 0.001). However, the long-to-short axis ratio did not differ significantly between the two groups (t = 0.49; p = 0.624) (Table 5).

**Table 5:** Comparison of Lymph Node Measurements According to Microvascular Flow Imaging Findings (n = 90) Data are presented as mean ± standard deviation (SD). An independent samples t-test was used for comparison. A p-value of <0.05 was considered statistically significant mm: millimeters

Variable	Vascularity Present (n = 81)	Vascularity Absent (n = 9)	Independent T-value	P-value
Long-axis diameter (mm)	17.1 ± 4.4	11.5 ± 2.8	4.18	<0.001
Short-axis diameter (mm)	7.1 ± 1.9	4.6 ± 1.3	4.73	<0.001
Long-to-short axis ratio	2.41 ± 0.52	2.50 ± 0.49	0.49	0.624

Diagnostic agreement analysis demonstrated moderate agreement between Color Doppler and Microvascular Flow Imaging for the assessment of mesenteric lymph node vascularity. Color Doppler demonstrated a sensitivity of 82.7%, a specificity of 100.0%, a positive predictive value of 100.0%, a negative predictive value of 39.1%, and an overall diagnostic accuracy of 84.4%. Agreement between the two imaging modalities was moderate, with a Cohen's kappa coefficient of 0.52 (95% CI: 0.34-0.70), and this agreement was statistically significant (Z = 5.84; p < 0.001) (Table 6).

**Table 6:** Diagnostic Agreement Between Color Doppler and Microvascular Flow Imaging in the Assessment of Mesenteric Lymph Node Vascularity (n = 90) Data are presented as diagnostic performance measures and agreement statistics. Cohen's kappa analysis with a Z-test was used to assess agreement. A p-value of <0.05 was considered statistically significant CI: confidence interval

Parameter	Value (95% CI)
Sensitivity	82.7% (95% CI: 72.7%-90.2%)
Specificity	100.0% (95% CI: 66.4%-100.0%)
Positive predictive value	100.0% (95% CI: 94.6%-100.0%)
Negative predictive value	39.1% (95% CI: 19.7%-61.5%)
Diagnostic accuracy	84.4% (95% CI: 75.3%-91.2%)
Cohen's kappa (95% CI)	0.52 (0.34-0.70)
Z statistic	5.84
P-value	<0.001

## Discussion

The present study included 90 pediatric patients, with the highest proportion belonging to the 6-10-year age group (43 {47.8%}), followed by 2-5 years (28 {31.1%}) and 11-15 years (19 {21.1%}). A slight male predominance was observed, with 51 (56.7%) men and 39 (43.3%) women. Abdominal pain was the most common presenting symptom, occurring in 77 (85.6%) patients, followed by fever in 41 (45.6%) and vomiting in 34 (37.8%). Grayscale ultrasonography demonstrated that most enlarged mesenteric lymph nodes were located in the periumbilical region (38 {42.2%}) and right iliac fossa (33 {36.7%}), while 79 (87.8%) nodes exhibited an oval morphology, and 80 (88.9%) showed the preservation of the central fatty hilum. These findings are comparable to those reported by Zu et al., who observed that reactive mesenteric lymph nodes retained benign ultrasonographic morphology with preserved hilar architecture and significantly larger nodal diameters in children with mesenteric lymphadenitis compared with healthy controls, reinforcing the importance of grayscale ultrasonography as the primary imaging modality for evaluating pediatric mesenteric lymphadenopathy [5]. The predominance of oval-shaped lymph nodes with preserved fatty hila in the present study further supports the benign inflammatory nature of mesenteric lymphadenopathy in most pediatric patients and highlights the continuing value of grayscale ultrasonography as the initial imaging modality before vascular assessment.

A major finding of the present study was the significantly greater detection of intranodal vascularity using Microvascular Flow Imaging compared with conventional Color Doppler. Vascularity was identified in 81 (90.0%) patients using Microvascular Flow Imaging compared with 67 (74.4%) patients using Color Doppler (p = 0.004). Moreover, Grade G3 vascularity was detected in 27 (30.0%) patients using Microvascular Flow Imaging compared with 13 (14.4%) using Color Doppler, whereas Grade G0 vascularity decreased from 23 (25.6%) to nine (10.0%). These observations are in agreement with the study by Zu et al., in which superb microvascular imaging detected vascular signals in 25/27 (92.6%) abnormal mesenteric lymph nodes compared with 23/27 (85.2%) using conventional Color Doppler flow imaging [5]. The authors further demonstrated that grayscale ultrasonography combined with superb microvascular imaging achieved a diagnostic sensitivity of 22/27 (81.5%), compared with 20/27 (74.1%) for grayscale ultrasonography combined with Color Doppler, indicating the superior capability of microvascular imaging for demonstrating low-velocity intranodal blood flow. A more recent prospective study by Zhu et al. similarly demonstrated that the addition of superb microvascular imaging significantly improved the diagnostic performance of grayscale ultrasonography in distinguishing pediatric mesenteric lymphadenitis from normal lymph nodes, supporting the growing clinical utility of advanced microvascular imaging techniques in pediatric abdominal ultrasound [14]. The enhanced visualization of low-velocity intranodal blood flow achieved with MVFI is likely attributable to its advanced clutter suppression algorithms, which preserve weak vascular signals while reducing motion artefacts. Consequently, MVFI may detect subtle inflammatory vascular changes that are not readily identified using conventional Color Doppler, thereby improving lesion characterization and diagnostic confidence.

The present study also demonstrated that patients with detectable vascularity on Microvascular Flow Imaging had significantly larger lymph node dimensions than those without vascularity. The mean long-axis diameter was 17.1 ± 4.4 mm in patients with vascularity compared with 11.5 ± 2.8 mm in those without vascularity (p < 0.001), while the corresponding short-axis diameters were 7.1 ± 1.9 mm and 4.6 ± 1.3 mm, respectively (p < 0.001). However, the long-to-short axis ratio did not differ significantly (p = 0.624), suggesting that nodal enlargement rather than nodal shape is more closely associated with inflammatory vascular activity. Similar observations were reported by Zu et al., who found significantly larger lymph node diameters in mesenteric lymphadenitis without significant differences in the long-to-short axis ratio between diseased and healthy children [5]. Likewise, Bayramoglu et al. evaluated 45 malignant, 72 inflammatory, and 146 normal pediatric lymph nodes using superb microvascular imaging and demonstrated that increased vascularity and vascularity index were closely associated with pathological lymph nodes, highlighting the value of microvascular imaging in characterizing lymph node vascular architecture beyond conventional grayscale assessment [17]. These findings suggest that vascular assessment should complement, rather than replace, conventional morphological evaluation, as combining grayscale characteristics with microvascular information provides a more comprehensive assessment of mesenteric lymph nodes.

Diagnostic agreement analysis in the present study demonstrated that Color Doppler achieved a sensitivity of 82.7%, a specificity of 100.0%, a positive predictive value of 100.0%, a negative predictive value of 39.1%, and an overall diagnostic accuracy of 84.4%, with moderate agreement between the two imaging techniques (Cohen's κ = 0.52; 95% CI: 0.34-0.70; p < 0.001). These findings support the complementary role of Microvascular Flow Imaging in routine pediatric ultrasonography. Ohno et al. similarly demonstrated that superb microvascular imaging consistently outperformed conventional Doppler imaging for depicting microvascular structures in pediatric hepato-gastrointestinal disorders [18]. In their series of 56 children undergoing 81 examinations, abnormal vascularity was visualized in 9/9 (100%) examinations of the appendix, 9/11 (81.8%) intestinal examinations, and 3/3 (100%) lymph node examinations, confirming the superior capability of microvascular imaging to detect slow-flow vascular signals without the need for contrast administration. Collectively, these findings indicate that Microvascular Flow Imaging provides additional clinically relevant vascular information beyond conventional Color Doppler and may improve diagnostic confidence in the evaluation of pediatric mesenteric lymphadenopathy.

From a practical perspective, MVFI can be incorporated into routine pediatric abdominal ultrasonography whenever enlarged mesenteric lymph nodes are identified on grayscale imaging. As the technique does not require intravenous contrast administration or substantially prolong examination time, it can be readily integrated into standard ultrasound protocols. The improved visualization of intranodal vascularity may facilitate the differentiation of reactive inflammatory lymph nodes from clinically significant pathology, reduce diagnostic uncertainty, and assist clinicians in selecting appropriate patient management while avoiding unnecessary additional imaging or surgical intervention.

Strengths and limitations

The present study has several strengths, including its prospective study design, standardized imaging protocol, and direct comparison of Color Doppler with Microvascular Flow Imaging in the evaluation of mesenteric lymph nodes in pediatric patients. The assessment of both grayscale morphological characteristics and vascularity patterns provided a comprehensive evaluation of lymph node status, while the use of a uniform vascularity grading system enhanced consistency in image interpretation. The standardization of image acquisition and vascularity grading before study initiation further improved the reproducibility of the imaging assessments.

However, certain limitations should be acknowledged. The study was conducted at a single tertiary care center with a relatively modest sample size, which may limit the generalizability of the findings. Only the largest representative lymph node from each patient was evaluated, which may not fully reflect the vascular characteristics of all enlarged mesenteric lymph nodes. Furthermore, histopathological confirmation was not available because tissue sampling is not routinely indicated or ethically justified in children with clinically suspected reactive mesenteric lymphadenopathy. Consequently, diagnostic performance was assessed using MVFI as the imaging reference standard rather than histopathological confirmation. Future multicenter studies with larger sample sizes and long-term clinical follow-up are warranted to validate these findings and establish standardized diagnostic criteria for the routine clinical application of MVFI in pediatric mesenteric lymphadenopathy.

## Conclusions

Microvascular Flow Imaging demonstrated superior performance compared with conventional Color Doppler in detecting intranodal vascularity in mesenteric lymph nodes among pediatric patients, enabling the improved visualization of low-velocity blood flow and the identification of higher vascularity grades while demonstrating moderate diagnostic agreement with conventional Color Doppler. When used as an adjunct to grayscale ultrasonography and conventional Color Doppler, Microvascular Flow Imaging provided additional vascular information that enhanced the characterization of mesenteric lymph nodes and may improve diagnostic confidence in children presenting with abdominal pain and suspected mesenteric lymphadenopathy. These findings support the incorporation of Microvascular Flow Imaging as a valuable adjunct to the routine ultrasonographic evaluation of pediatric mesenteric lymph nodes; however, larger multicenter prospective studies are warranted to validate these findings, establish standardized imaging protocols, and further define the role of MVFI in routine pediatric abdominal ultrasonography.
